# Inhibition of LRRK2-Rab10 Pathway Improves Secondary Brain Injury After Surgical Brain Injury in Rats

**DOI:** 10.3389/fsurg.2021.749310

**Published:** 2022-01-05

**Authors:** Jie Li, Muyao Wu, Yating Gong, Jiafeng Tang, Jinchao Shen, Li Xu, Baoqi Dang, Gang Chen

**Affiliations:** ^1^Department of Intensive Care Unit, Zhangjiagang Traditional Chinese Medicine Hospital, Nanjing University of Chinese Medicine, Suzhou, China; ^2^Department of Rehabilitation, Zhangjiagang Traditional Chinese Medicine Hospital, Nanjing University of Chinese Medicine, Suzhou, China; ^3^Department of Anesthesiology, Zhangjiagang Traditional Chinese Medicine Hospital, Nanjing University of Chinese Medicine, Suzhou, China; ^4^Department of Neurosurgery and Brain and Nerve Research Laboratory, The First Affiliated Hospital of Soochow University, Suzhou, China

**Keywords:** LRRK2, Rab10, surgical brain injury, apoptosis, lysosomal hydrolase

## Abstract

Leucine-rich repeat kinase 2 (LRRK2) is considered as a potential target for the treatment of Parkinson's disease. This protein is expressed in the brain and has been associated with various diseases and lysosomal maintenance. Rab10 is a member of the Rab protein GTPase family that has been recently shown to be a kinase substrate of LRRK2. In addition, LRRK2 and its kinase substrate Rab10 constitute a key stress response pathway during lysosomal overload stress. This study aimed to investigate the potential role and mechanism underlying LRRK2 and its kinase substrate Rab10 involving surgical brain injury (SBI). One hundred and forty-four male Sprague-Dawley rats were examined using an SBI model, and some had received the LRRK2-specific inhibitor PF-06447475. Thereafter, western blotting, immunofluorescence, brain water content analysis, neuronal apoptosis assay, and neurological score analysis were conducted. The results showed that after SBI, LRRK2 and phosphorylated Rab10 (p-Rab10) expression in neuronal cells were upregulated, and administration of PF-06447475 significantly reduced neuronal apoptosis, neuroinflammation, and brain water content 12 h after SBI and improved neurological deficit 72 h after SBI, which is related to the decreased expression of LRRK2 and p-Rab10, and the lessening of lysosomal overload stress. Our research suggests that the inhibition of LRRK2 can effectively interfere with the role of p-Rab10 in promoting the secretion of lysosomal hydrolase in lysosomal overload stress after SBI, thereby reducing neuronal apoptosis and inflammation after SBI and playing a major role in brain protection.

## Introduction

Surgical brain injury (SBI) may be the inevitable sequelae of neurosurgery, leading to intracranial hematoma and brain edema (1). It often occurs in neurosurgery during removal of the surrounding brain tissue and is caused by hemorrhage, retraction, incision trauma, and electrocoagulation (2, 3). SBI can lead to cerebral edema, apoptosis, inflammation and other pathological processes around the surgical site, and reduce a patient's quality of life (4). Only a few studies on the mechanism of brain cell damage after SBI have been conducted, and thus no effective treatment plan has been established to date. Therefore, the mechanism and time course of secondary injury after SBI can be used as an important reference and guide for targeted therapy.

Lysosomes are cytoplasmic vesicles containing acidic lysosomal cathepsins and are surrounded by a single membrane. Lysosomes are ubiquitously distributed in all mammalian cells, except for red blood cells (5). Lysosomes play an important role in the metabolic cycle of substances inside and outside cells. Substances contained in phagocytic vesicles that fuse with lysosomes are degraded into small molecules after interacting with lysosomal hydrolases, thereby maintaining normal physiology within the cell (6). In an earlier study using a rat model of focal cerebral ischemia, lysosomal autophagy in neuronal cells is activated, clearing damaged organelles, which induces lysosomal overload stress and eventually leading to nerve death (7). The Levine/Vannucci mouse model was used to simulate perinatal hypoxic-ischemic brain damage. Eventually, hypoxia-ischemia induced lysosomal overload stress, which in turn triggered neuronal cell death (8). Lysosomal overload stress induces the translocation of leucine-rich repeat kinase 2 (LRRK2) to lysosomes where it is activated, which in turn stabilizes Rab8 and Rab10 *via* phosphorylation (9).

LRRK2 is a large multi-domain leucine kinase with multiple enzyme binding and protein binding sites. It is expressed in the brain, kidney, lung, heart, liver as well as immune tissues and plays a major role in regulating nervous system functions (10, 11). LRRK2 is widely expressed in neurons, glial cells and immune cells, and is involved in vesicle transport, neurotransmitter release, synaptic plasticity, Golgi and mitochondrial function, immune response and other cellular functions and pathways (9, 12). LRRK2 is involved in many human diseases and is the most important mutant gene in familial and sporadic Parkinson's disease (PD) (13). The protein expression level of LRRK2 is related to apoptosis, secretion of pro-inflammatory factors, autophagy disorders, and defects in oxidative damage (14–16), processes that are associated with increased brain damage after TBI (17). LRRK2 is shown to be involved in the endolysosomal vesicular trafficking which involves the Rab family of small GTPases (9).

Rab GTPases are the master regulators of vesicular transport proofreading, organelle biogenesis and cellular homeostasis. Rab10 is a member of Rab GTPases and has the function of TGN-to-plasma membrane trafficking, GLUT4 trafficking, ciliogenesis, phagosome maturation (18). Recent studies have shown that a subset of Rab GTPase proteins serves as biological substrate of LRRK2, which can directly phosphorylate many Rab proteins (including Rab10) (19). LRRK2 can directly phosphorylate the effect switch II motif, including Rab10 (Thr73). Research studies on LRRK2 and Parkinson's disease have shown that Rab10 can be effectively phosphorylated by LRRK2, thus proving the direct relationship between this kinase and substrate (20). Under the condition of lysosomal overload stress induced by chloroquine (CQ), Rab10 is phosphorylated by overexpressed LRRK2, which promotes the release of lysosome-secreted cathepsin to the outside of the cell that in turn triggers apoptosis (9, 21, 22). Therefore, this study aimed to explore the expression and mechanism of the LRRK2-Rab10 stress response pathway in neurons of a rat SBI model to provide a theoretical basis for the clinical treatment and new drug development for SBI.

## Materials and Methods

### Animals

**Figure 1:**
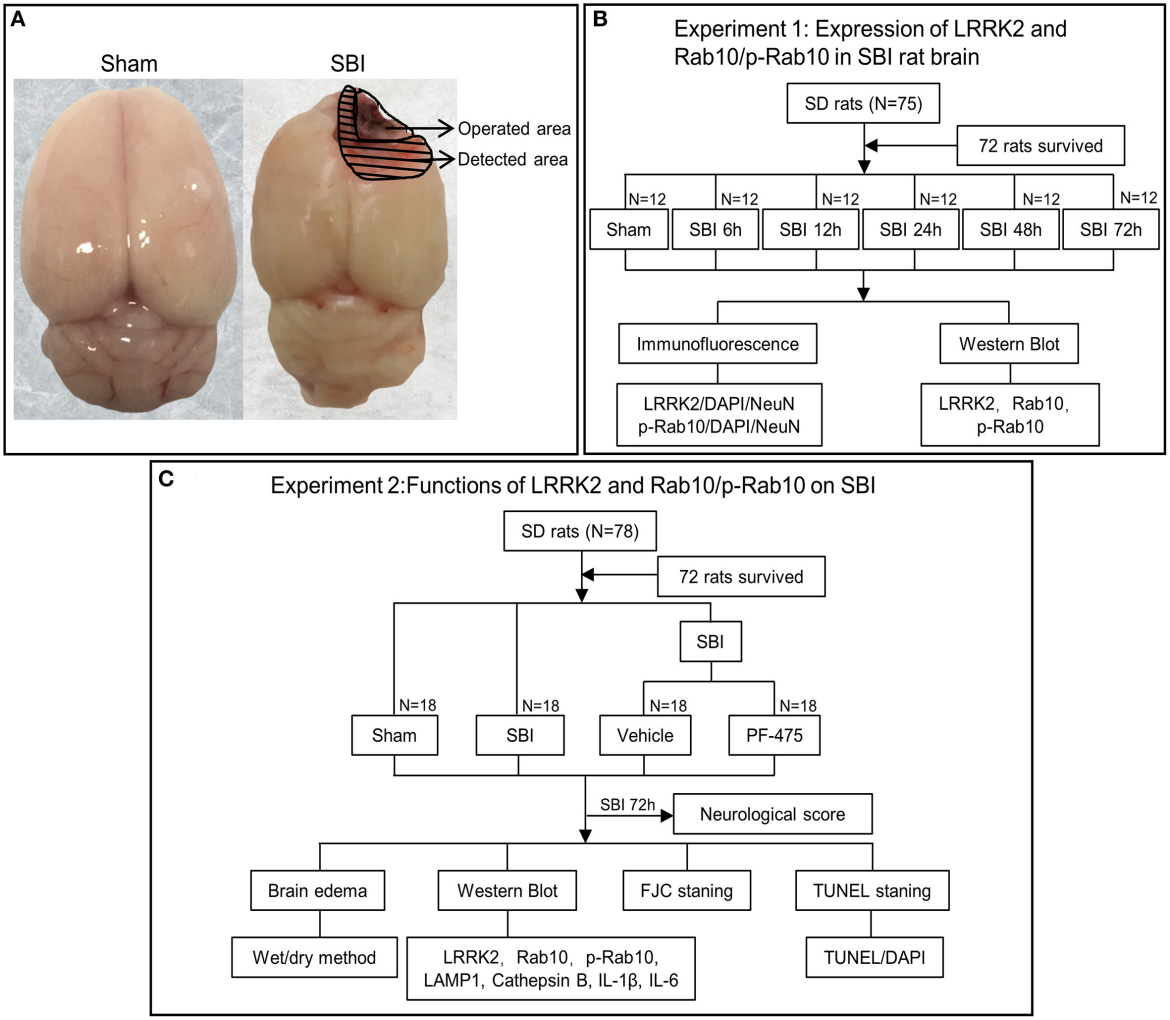
Experimental design. **(A)** Brain tissue was obtained from the same site (peripheral lesion) in the SBI group and the Sham group for measurement. **(B)** Experiment 1 was to prove the expression level and location of LRRK2, Rab10, p-Rab10 after SBI, and to determine the appropriate time point for Experiment 2. **(C)** Experiment 2 aimed to observe the effect of LRRK2/p-Rab10 on early brain injury after SBI and explore its potential mechanism.

In this study, 153 male Sprague-Dawley rats with a weight between 350 and 400 grams were obtained from the Zhaoyan (Suzhou) New Drug Research Center. Only 144 of the 153 rats were used for statistical analysis. In experiment 1 (Figure 1B): 36 rats (6 rats in each group) were assessed with WB and IF staining was performed on the other 36 rats (6 rats in each group). In experiment 2 (Figure 1C): 24 rats (6 rats in each group) were assessed with WB, TUNEL and FJC staining; 24 rats (6 rats in each group) were assessed in terms of brain edema, and the other 36 rats (6 in each group) were used to assess neurological score before they were sacrificed. All experimental protocols were approved by the Institute of Animal Care Committee of Zhangjiagang Traditional Chinese Medicine Hospital and were implemented following the guidelines of the National Institutes of Health Guidelines for the Care and Use of Experimental Animals.

### SBI Model and Drug Injection

The SBI model has been described in previous studies (23, 24). SBI surgery consists of partial removal of the right frontal lobe. This procedure uses 40 mg/kg pentobarbital intraperitoneal injection to anesthetize rats. Upon anesthesia, the skin was cut and a square cranial window (5 mm edge) was made by drilling into the anterior right direction of the bregma. The dura mater was then cut open and the underlying right frontal lobe was isolated. Partial right frontal lobectomy was conducted (2 mm lateral to the sagittal suture and 1 mm proximal to the coronal suture). The frontal lobe was flushed with filler and saline solution to maintain hemostasis, then the skin was sutured. Sham group rats included craniotomy and flap replacement only, without any dural incisions (Figure 1A). After the operation, the animals were sacrificed at different time points, as shown in the following experiment.

The treatments consisted of the Sham group, SBI group, SBI + Vehicle group, and SBI + PF-475 group. The LRRK2 inhibitor PF-475 (Sellcek, USA) was dissolved in dimethyl sulfoxide (DMSO) and then diluted with 0.9% saline to a final concentration of <1% in DMSO. Later, PF-475 (5 mg/kg) or vehicle (DMSO in saline) was intraperitoneally injected at 1 h post-SBI modeling (25).

### WB Analysis

WB analysis was conducted as previously described (26). First, the samples were homogenized in RIPA lysis buffer containing a protease inhibitor (Beyotime, China), followed by 13,000 g centrifugation at 4°C for 20 min to extract the proteins from the damaged areas. After determining total protein concentration using a Pierce™ BCA protein detection kit (Thermo Fisher, USA), separation of equivalent extracted proteins was performed by electrophoresis on a 10% sodium dodecyl sulfate polyacrylamide gel (Beyotime), followed by transferring the bands onto a PVDF membrane (Millipore, USA). The membrane was then blocked with a blocking buffer containing 5% skim milk for 2 h at room temperature, and then hybridized with the following primary antibodies at 4°C overnight: rabbit anti-LRRK2 (1:10,000, Abcam, UK), rabbit anti-Rab10 (phosphate T73) (1:1,000, Abcam), rabbit anti-Rab10 (1:1,000, Abcam), rabbit anti-LAMP1 (1:1,000, Abcam), rabbit anti-Cathepsin B (1:1,000, Abcam), anti-IL-1β (1:5,000, Abcam), anti-IL-6 (1:1,000, ABclonal, China). Mouse anti-β-actin (1:10,000, Sigma, USA) was used as internal loading control. The membranes were then hybridized to horseradish peroxidase-conjugated secondary antibodies, goat anti-rabbit IgG-HRP (Invitrogen, USA) for 2 h at 4°C. WBs were assessed using an Immobilon™ western chemiluminescent HRP substrate (Millipore) and visualized with a LUMINESCENT IMAGE ANALYZER (GE Healthcare, Sweden). All data were assessed using ImageJ software (National Institutes of Health, Bethesda, MD, USA).

### Immunofluorescence Staining

Double immunofluorescence staining was conducted as described elsewhere (27). After washing thrice with 0.3% Triton in phosphate-buffered saline (PBS) to permeabilize the cell membrane, the frozen brain sections (15 μm thick) were blocked with an immunostaining blocking buffer (Beyotime) at room temperature for 1 h and then incubated at 4°C overnight using the following primary antibodies: rabbit anti-Rab10 (phospho T73) (1:200, Abcam), rabbit anti-LRRK2 (1:200, Abcam), and mouse anti-NeuN (1:300, Cell Signaling, USA). After incubating with Alexa Fluor 488 donkey anti-mouse IgG antibody (1:800, Invitrogen) and Alexa Fluor 555 donkey anti-rabbit IgG antibody (1:800, Invitrogen) at room temperature for 1 h, the sections were stained for 10 min with 4′, 6-dididia-2-phenylindolediamine hydrochloride (DAPI), and were assessed thereafter under a fluorescence microscope (OLYMPUS, U-RFL-T, Japan).

### Brain Edema

Brain water content was evaluated using the wet-dry method (28). After the rat brain was dissected, it was divided into ipsilateral and contralateral hemispheres, and the wet weight was immediately weighed. The sample was then placed in an oven at 100°C for 48 h and weighed dry weight. The percentage (%) of brain water content was calculated as [(Wet weight-dry weight)/(Wet-weight)] × 100%.

### TUNEL Staining

Apoptosis was detected using TUNEL staining kit (Beyotime). The frozen brain sections were placed in PBS for 15 min, and then transferred to a protease K working solution and incubated for 5 min. After three washes in PBS, the brain sections were covered with DNA labeling solution in a dark, humidified box for 1 h. Then, the brain sections were incubated with the antibody solution in a dark, damp box for 30 min. Following that, the brain sections were air-dried and then counterstained with DAPI. The images were observed using an epifluorescence microscope (OLYMPUS, Japan).

### FJC Staining

FJC staining was performed following the manufacturer's instructions (Biosensis, South Australia). The brain tissue sections were rinsed with distilled water for around 2 min. Then, the sections were incubated in a solution consisting of distilled water and potassium permanganate (9:1 ratio) for 10 min. After incubated in a solution consisting of distilled water and FJC Solution (9:1 ratio) for 30 min, the sections were rinsed with distilled water thrice, dried for 5 min at 50–60°C, and then immersed in xylene for 5 min. Lastly, the sections were mounted with neutral resin and viewed under an epifluorescence microscope (OLYMPUS). We reported the total number of FJC-positive cells as the average number of FJC-positive cells per field of view. All procedures, consisting of sectioning, on-site selection, and cell counting, were conducted by investigators who were blinded to the animals' conditions.

### Neurological Scoring

Neurological deficiency was assessed at 72 h after SBI with the modified Garcia score (29, 30). Briefly, this includes seven parameters: spontaneous activity, body proprioception, response to tentacle touch, symmetry of limb movement, lateral bending, forelimb walking, and climbing ability. Each subtest has scores ranging from 0 to 3, and the maximum combined score was 21 (no neurological deficit).

### Statistical Analyses

All data were expressed as the mean ± SD. GraphPad Prism 8.0 software (San Diego, CA, USA) was employed for statistical analysis. The Kolmogorov–Smirnov-test was used to assess the distribution of each dataset. Two data groups showing normal distributions were compared by the two-tailed, unpaired, Student's *t*-test, while the Mann–Whitney *U*-test was employed for non-parametric data. Differences with *P* < 0.05 were considered statistically significant. The experiment was performed by strictly following the blind method. All samples in the experiment were coded by independent investigators. Blinded data analysis was used for all sample types.

## Results

### Temporal Expression of LRRK2, p-Rab10, and Rab10 in the Brain After SBI

**Figure 2:**
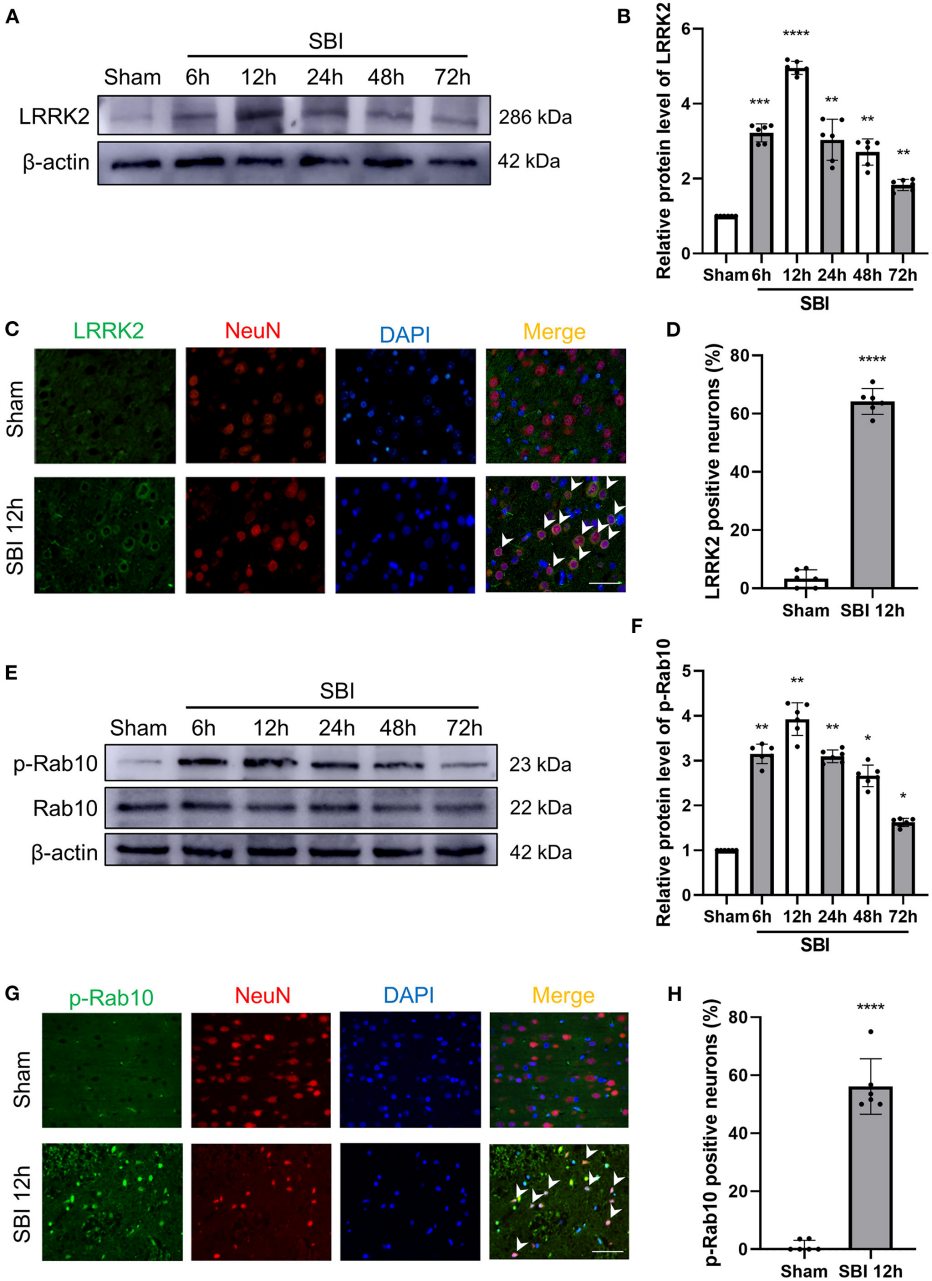
Post-SBI protein expression level and immunofluorescent staining of LRRK2 and p-Rab10 in peri-injury cortex. **(A,B)** LRRK2 protein level in brain after SBI. **(C,D)** Double immunofluorescence analysis of LRRK2 (green) in neurons (red) near the damaged area after SBI. **(E,F)** Rab10/p-Rab10 protein level in brain after SBI. **(G,H)** Double immunofluorescence analysis of p-Rab10 (green) in neurons (red) near the damaged area after SBI. Nuclei were labeled with DAPI (blue). Bar = 50 μm. Data = mean ± SD, *N* = 6. ^*^P < 0.05, ^**^P < 0.01, ^***^P < 0.005, ^****^P < 0.001 vs. Sham; ns *P* > 0.05 vs. Sham.

In order to clarify the expression of LRRK2 and Rab10 after SBI, 72 rats (72 of 75 rats survived) were randomly assigned to seven groups by computer-based randomization to establish the time course (6, 12, 24, 48, and 72 h) post-SBI in Experiment 1. No significant differences were observed in the weight, food consumption, and exercise ability of the rats in every group. The tissues around the damaged area were used western blot (WB) analysis to assess LRRK2, Rab10, and p-Rab10 expression in SBI rat brains (Figure 1B). The results showed that the LRRK2 protein levels in the SBI group began to increase after SBI and peaked at 12 h, and then the protein levels began to decrease (Figures 2A,B). Similar to LRRK2, p-Rab10 expression began to increase after SBI and peak at 12 h, followed by a decline. Rab10 protein levels did not significantly change with the passage of time in SBI (Figures 2E,F). These results indicated that LRRK2 and p-Rab10 reached the peak expression at 12 h after SBI, and LRRK2 inhibition was performed 12 h after SBI in Experiment 2.

### Expression of LRRK2 and p-Rab10 in Neuronal Cells After SBI

According to the temporal expression of LRRK2 and p-Rab10 after SBI, the peak expression 12 h was selected for double immunofluorescence (IF). The expression and position of LRRK2 and p-Rab10 were further evaluated by IF staining of neuron-labeled NeuN at 12 h after SBI. The results of IF analysis coincided with the WB findings. Compared to the Sham group, the number of neuron that were positively stained with LRRK2 (Figures 2C,D) and p-Rab10 (Figures 2G,H) significantly increased in the SBI 12 h group.

### Effect of PF-06447475 Intervention on LRRK2 and p-Rab10 Protein Expression After SBI

**Figure 3:**
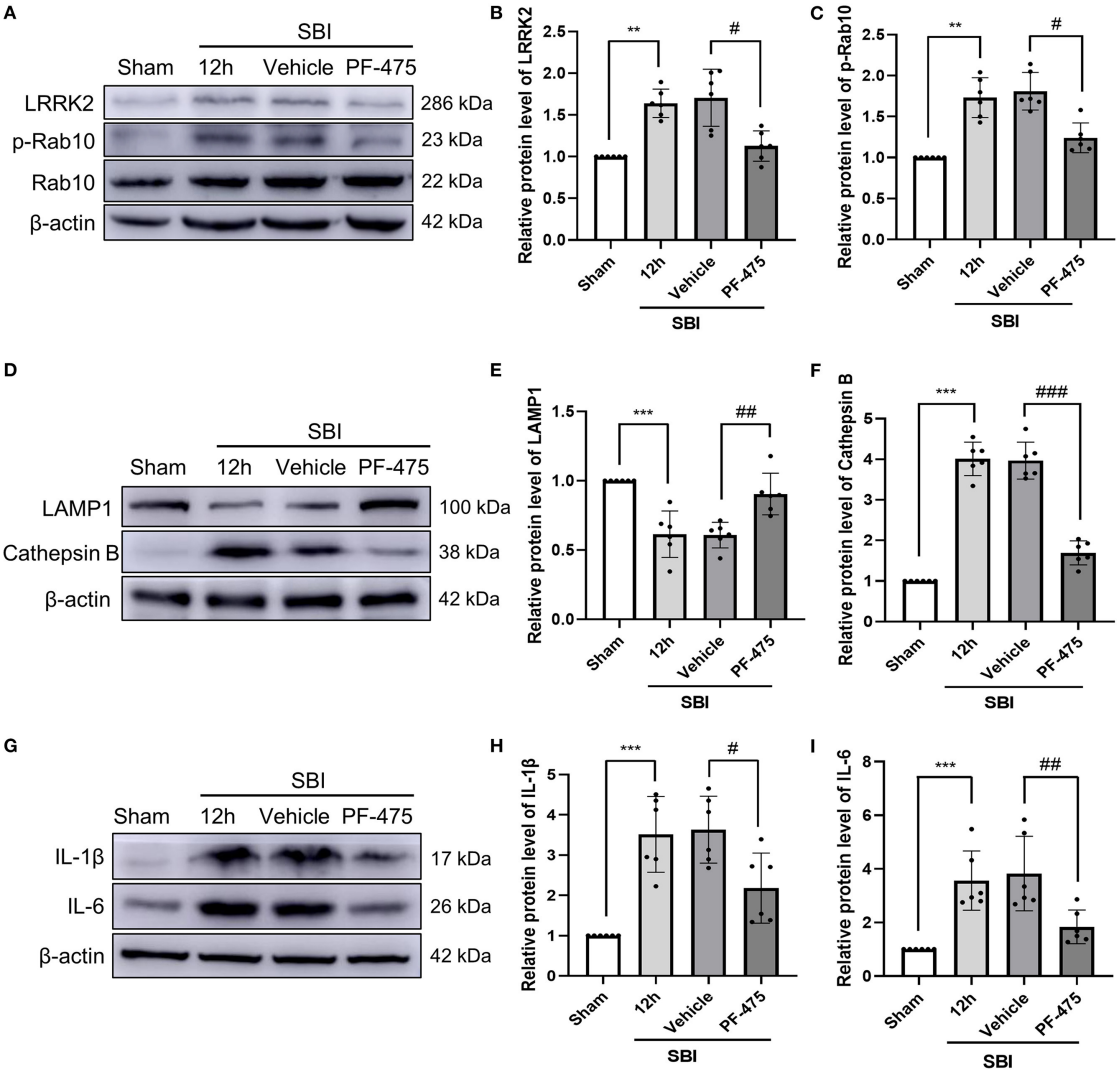
Changes in protein levels of LRRK2 and p-Rab10, lysosomal damage and inflammation in damaged brain tissue after injecting PF-475. Western blot was used to detect the expression of LRRK2 **(A,B)** and Rab10/p-Rab10 **(A,C)**, LAMP1 **(D,E)** and Cathepsin B **(D,F)**, IL-1β **(G,H)**, and IL-6 **(G,I)** in Sham, SBI, SBI + Vehicle and SBI + PF-475 12 h after SBI. Protein levels were quantified with Image J software. Data = means ± SD. *N* = 6. ^**^P < 0.01, ^***^P < 0.005 vs. Sham, ^#^*P* < 0.05, ^##^*P* < 0.01, ^###^*P* < 0.005 vs. SBI + PF-475.

To establish whether inhibiting LRRK2 decreases the degree of brain injury after SBI, PF-06447475 (PF-475), a specific inhibitor of LRRK2, was injected intraperitoneally 1 h after SBI. Seventy-two rats (72 of 78 rats survived) were randomly assigned to four groups based on computer randomization, which consisted of the Sham, SBI 12 h, SBI + Vehicle, and SBI + PF-475 groups. The rats were sacrificed and then damaged brain tissues were collected at 12 h after SBI. The WB results showed that compared with the Sham group, the protein expression of LRRK2 (Figures 3A,B) and p-Rab10 (Figures 3A,C) in the SBI group significantly increased. After injecting PF-475, the protein expression of LRRK2 and p-Rab10 significantly decreased compared with the SBI + Vehicle group.

### PF-06447475 Injection Improves Lysosomal Overload Stress and Inflammation After SBI

Lysosome associated membrane protein 1 (LAMP1) and lysosomal Cathepsin B were used to detect lysosomal damage. The protein expression of LAMP1 in the SBI group was decreased compared with the Sham group. After PF-475 injection, the protein expression of LAMP1 significantly increased compared with the SBI + Vehicle group (Figures 3D,E). In contrast, Cathepsin B protein expression in the SBI group was increased compared with that in the Sham group. After PF-475 injection, its expression was significantly decreased (Figures 3D,F). These results suggested that lysosome damage is aggravated by lysosome overload stress after SBI, and PF-06447475 can alleviate this damage. At the same time, the results showed that the expression of IL-1β (Figures 3G,H) and IL-6 (Figures 3G,I) in SBI 12 h group was higher than that in the Sham group, but decreased after PF-475 injection. These results indicated that SBI can aggravate the inflammation surrounding the injured brain tissue, and PF-475 injection can improve the inflammatory response after SBI.

### Inhibition of LRRK2 Ameliorates Neuronal Apoptosis and Necrosis After SBI

**Figure 4:**
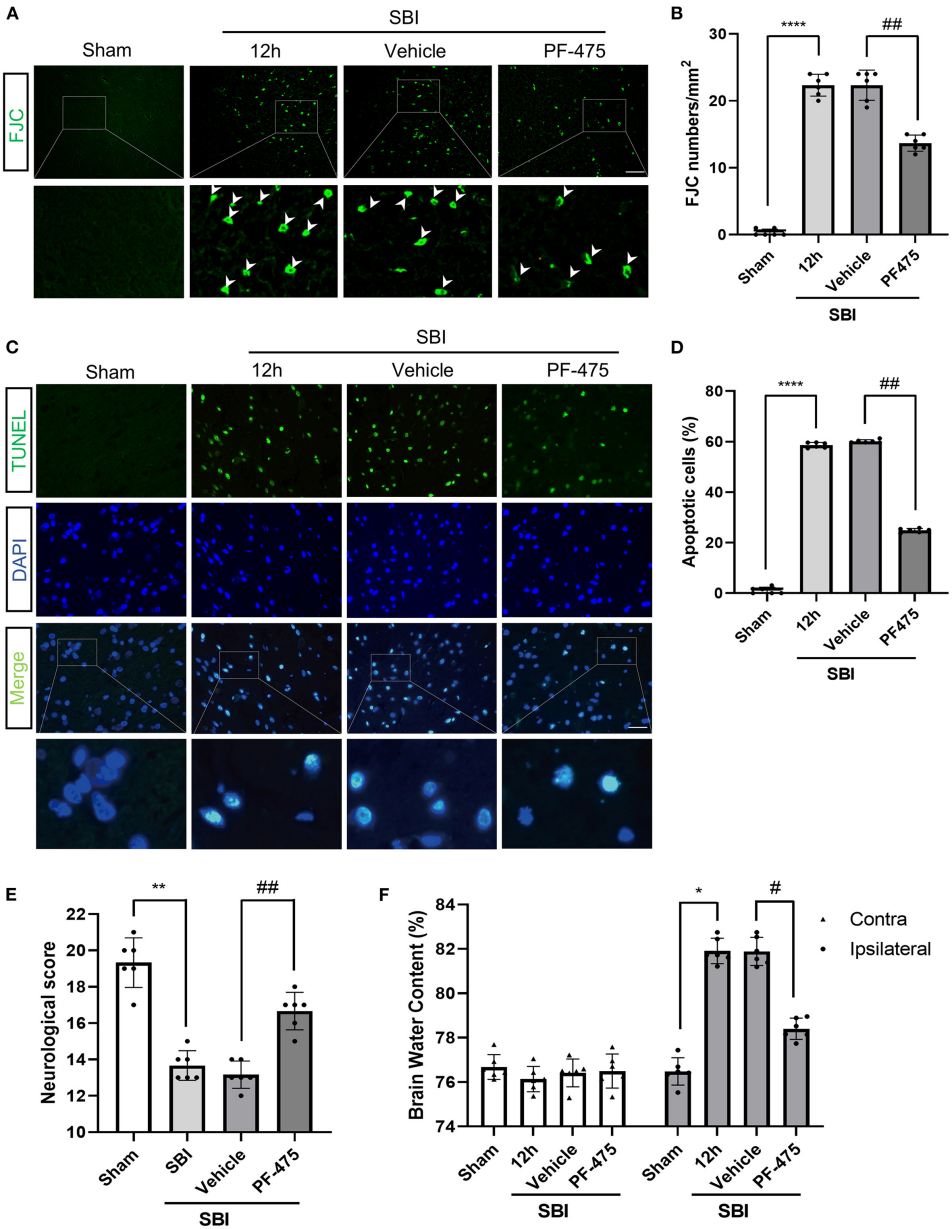
Effects of PF-475 intervention on cortex injury 12 h after SBI. **(A,B)** Neuron necrosis was detected by FJC staining. FJC-positive cells/mm2 was quantified. **(C,D)** Neuronal apoptosis was detected with the TUNEL assay. TUNEL-positive neurons were quantified. Bar = 50 μm, *N* = 6. **(E)** Neurological behavioral scores in SBI rats after PF-475 intervention. **(F)** The brain water content of the bilateral hemispheres of the different groups was measured using the wet-dry method. Data = mean ± SD, *n* = 6. ^*^P < 0.05, ^**^P < 0.01, ^****^P < 0.001 vs. Sham; ^#^*P* < 0.05, ^##^*P* < 0.01 vs. SBI + PF-475.

Terminal deoxynucleotidyl transferase-mediated dUTP nick end-labeling (TUNEL) staining was performed to determine neuronal apoptosis, and fluorescent jade C (FJC) staining was performed to determine neuronal necrosis. Apoptosis (Figures 4A,B) and necrosis (Figures 4C,D) of neurons were more extensive in the SBI 12 h group compared to the Sham group. After PF-475 treatment, the degree of apoptosis and necrosis was significantly lower than the SBI + Vehicle group. These results showed that inhibiting LRRK2 with PF-475 could ameliorate the neuronal apoptosis and necrosis after SBI.

### Effects of PF-06447475 Administration on Neurological Scores and Brain Edema After SBI

In order to verify the effect of PF-475 on brain edema in SBI rats, wet-dry method was used to detect the change of brain water content in different groups. Twelve hours after SBI, PF-475 treatment significantly reduced the brain edema in the injured hemispheres (*P* < 0.05), whereas the brain edema in the contra-lateral hemispheres did not significantly change (*P* > 0.05, Figure 4F). In parallel, neurological testing was performed in all groups prior to decollation. The injection of PF-475 significantly improved neurological scores at 72 h after SBI (*P* < 0.05, Figure 4E).

## Discussion

Our study assessed the expression of LRRK2 in brain injury post-surgery and investigated the potential mechanism using a rat SBI model. The results showed that endogenous LRRK2 and p-Rab10 expression increased following SBI. Inhibition of LRRK2 using PF-06447475 resulted in a reduction in neuronal apoptosis, inflammatory response, and brain edema, improvement of neurological deficits, and decrease in p-Rab10 expression and lysosomal overload stress. These observations suggest that LRRK2 is involved in SBI, at least in part, through the stress response pathway that is composed of LRRK2 and its kinase substrate Rab10.

To date, research on LRRK2 in brain tissues has mainly focused on PD and glial cell inflammation. Previous studies have revealed that reducing the kinase activity of LRRK2 decreases neuronal toxicity as well as neuronal apoptosis (31). In brain injury induced by TBI, LRRK2 protein expression is enhanced in the *in vitro* model, and the HIF-1α-dependent reverse transcription of LRRK2 is activated, causing brain tissue damage, cell death, and increased inflammation (32). In the rat TBI model, the endogenous expression of LRRK2 is increased, and p38/Drosha signaling is used to promote the aggravation of secondary brain injury, resulting in increased neuronal apoptosis, brain edema, and neurological deficit (17). In secondary brain injury using a rat intracerebral hemorrhage (ICH) model, it was observed that LRRK2 expression in the brain tissues after ICH had significantly increased, which in turn exacerbates the inflammatory response that is involved in secondary brain injury of ICH, thereby aggravating brain edema, blood–brain barrier damage, as well as cell death (33).

**Figure 5:**
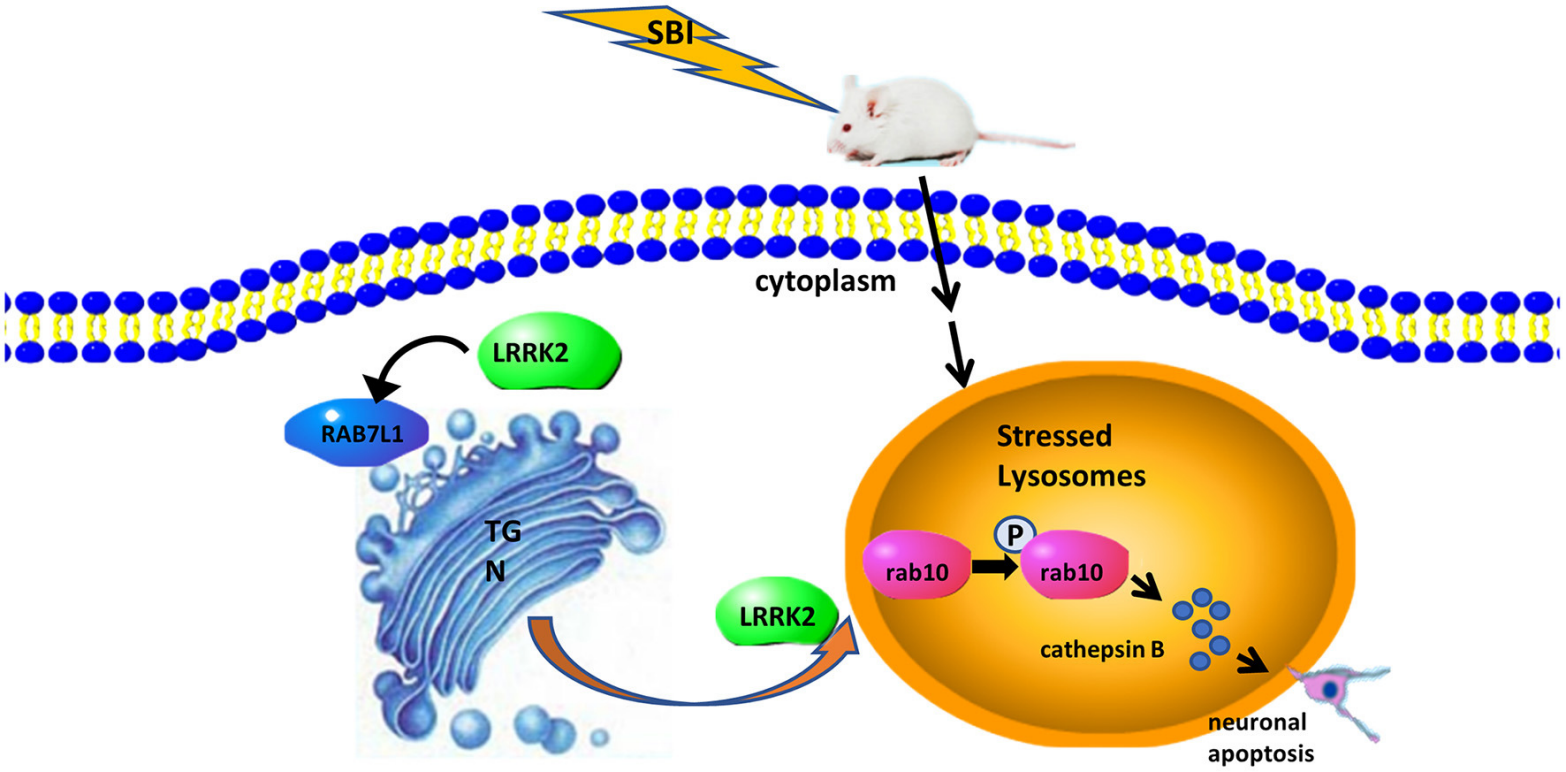
The potential mechanism of LRRK2-Rab10 stress response pathway-mediated neuronal apoptosis after SBI. SBI induces lysosomal overload stress in neurons and results in a rapid increase in endogenous LRRK2 expression, which can activate the Rab10 phosphorylation pathway and allow it to specifically accumulate in lysosomes. Overexpressed p-Rab10 promotes excessive lysosomal secretion of lysosomal cathepsins in the neurons, leading to destruction of the plasma membrane, cell lysis, ultimately neuronal apoptosis.

In this study, we focused on the mechanism of LRRK2 effect and its kinase substrate Rab10 on the brain injury in the SD rat SBI model. The results showed that in the secondary brain injury after SBI, the level of LRRK2 and p-Rab10 was increased, as well as the inflammation, apoptosis and necrosis of the surrounding brain tissue were aggravated. This may be due to the lysosomal autophagy function of neuron cells in the damaged brain tissue and induce lysosomal overload stress by clearing the damaged organelles. It was documented that the application of LRRK2 kinase inhibitor resulted in the downregulated protein expression of LRRK2 and p-Rab10 in the primary neurons of rats treated with rotenone (34, 35). Activating LRRK2 can phosphorylate its kinase substrate, Rab10, and cause its specific accumulation in lysosomes. Overexpression of phosphorylated Rab10 causes lysosomes to secrete cathepsin (mature cathepsins B and D and intermediate active cathepsin D) into extracellular matrix (36). If sufficient amounts of lysosomal cathepsins are released from the lysosome, the plasma membrane will be damaged, causing cell lysis (37, 38). The enzyme activity of lysosomal protease cathepsin B was observed to increase throughout the hippocampus 3–5 days after transient ischemia in monkeys. Using a cathepsin B-specific inhibitor was associated with saving 67% of CA1 neurons from undergoing delayed neuron death. The surviving neurons that were rescued by blocking lysosomal activity exhibited the reduce cathepsin B immunoreactivity (39). These observations imply that cathepsin B release is critical to the occurrence of ischemic neuron death. Our results showed that the expression of Cathepsin B was increased in the injured brain tissue after SBI, while LAMP1 expression was decreased, leading to lysosome degradation and increased apoptosis and necrosis. After inhibiting LRRK2 with PF-06447475, the expression of Cathepsin B was significantly decreased compared with that after SBI, while the expression of LAMP1 was increased, leading to less lysosomal damage and less apoptosis and necrosis. In summary, these findings support our hypothesis that elevated levels of LRRK2 protein activate the phosphorylation of Rab10 and promote excessive secretion of lysosomal cathepsin B, leading to neuronal cell apoptosis, aggravating the degree of secondary brain injury in SBI rats (Figure 5). Inhibition LRRK2 with PF-06447475 may reverse this apoptosis and necrosis by inhibiting the release of cathepsin B, reduce inflammation and brain edema, and improve neurological deficits.

The current study has a number of limitations. LRRK2 is also expressed in glial cells and thus the activation of microglial cells enhanced by endogenous mutant LRRK2 could intensify the release of neurotoxic cytokines by microglial cells, which in turn increases the direct toxicity of mutant LRRK2 that is expressed by neurons (40). Strong LRRK2 induction in microglia was detected in the mouse neuroinflammation model and inflammation enhanced LRRK2 activity and expression. LRRK2 inhibitors attenuate pro-inflammatory cytokines production in activated microglia in culture, thus reducing brain neuroinflammation in Parkinson's disease (41). Our results also found that inhibition of LRRK2 alleviated neuroinflammation after SBI. However, whether the secretion of pro-inflammatory factors by glial cells due to the overexpression of LRRK2 contributes to secondary brain injury after SBI and its underlying mechanism remains unclear. We can focus on these directions in the following research.

## Conclusion

The LRRK2-Rab10 stress response pathway regulates the development of secondary brain injury after SBI. Inhibition of LRRK2 using PF-06447475 resulted in the decrease of LRRK2 and p-Rab10 expression, a reduction in neuronal apoptosis and brain edema, and the improvement of neurological deficits. It was inferred that LRRK2 may be a key physiologically active substance that is expressed after SBI brain injury that may be utilized as a therapeutic target for secondary brain injury after SBI.

## Data Availability Statement

The raw data supporting the conclusions of this article will be made available by the authors, without undue reservation.

## Ethics Statement

The animal study was reviewed and approved by the Institute of Animal Care Committee of Zhangjiagang Traditional Chinese Medicine Hospital.

## Author Contributions

BD and GC contributed to conception and design of the study. YG, JT, and JS organized the database. JL and MW performed the statistical analysis. JL wrote the first draft of the manuscript. LX and MW wrote sections of the manuscript. All authors contributed to manuscript revision, read, and approved the submitted version.

## Funding

This work was supported by Suzhou Gusu Health Personnel Training Project (GSWS2020104), Zhangjiagang Health Personnel Training Project (ZJGWSRC202003), Zhangjiagang Municipal Health Commission Youth Science and Technology Project (ZJGQNKJ202012), and Zhangjiagang Municipal Science and Technology Pillar Plan (ZKS1936).

## Conflict of Interest

The authors declare that the research was conducted in the absence of any commercial or financial relationships that could be construed as a potential conflict of interest.

## Publisher's Note

All claims expressed in this article are solely those of the authors and do not necessarily represent those of their affiliated organizations, or those of the publisher, the editors and the reviewers. Any product that may be evaluated in this article, or claim that may be made by its manufacturer, is not guaranteed or endorsed by the publisher.
